# Corporal diagnostic work and diagnostic spaces: clinicians’ use of space and bodies during diagnosis

**DOI:** 10.1111/1467-9566.12233

**Published:** 2015-02-13

**Authors:** John Gardner, Clare Williams

**Affiliations:** ^1^ Science and Technology Studies Unit (SATSU)Department of SociologyUniversity of YorkUK; ^2^ Department of Sociology and CommunicationsBrunel UniversityLondonUK

**Keywords:** diagnosis, body work, embodiment, neurosciences, space

## Abstract

An emerging body of literature in sociology has demonstrated that diagnosis is a useful focal point for understanding the social dimensions of health and illness. This article contributes to this work by drawing attention to the relationship between diagnostic spaces and the way in which clinicians use their own bodies during the diagnostic process. As a case study, we draw upon fieldwork conducted with a multidisciplinary clinical team providing deep brain stimulation (DBS) to treat children with a movement disorder called dystonia. Interviews were conducted with team members and diagnostic examinations were observed. We illustrate that clinicians use communicative body work and verbal communication to transform a material terrain into diagnostic space, and we illustrate how this diagnostic space configures forms of embodied ‘sensing‐and‐acting’ within. We argue that a ‘diagnosis’ can be conceptualised as emerging from an interaction in which space, the clinician‐body, and the patient‐body (or body‐part) mutually configure one another. By conceptualising diagnosis in this way, this article draws attention to the corporal bases of diagnostic power and counters Cartesian‐like accounts of clinical work in which the patient‐body is objectified by a disembodied medical discourse.

## Introduction

In 2003 deep brain stimulation (DBS) was approved as a means of managing dystonia, a neurological disorder that in severe cases is characterised by painful, crippling body postures. By 2010 approximately 1000 people worldwide had received DBS for dystonia, most of whom were adults with ‘primary’ dystonia, in which dystonia is the only neurological pathology. Generally these patients respond well to DBS: as DBS reduces their dystonic movements, they experience a marked functional gain.

Within the last five years a few centres worldwide have begun to offer DBS to people with secondary dystonia. Here, dystonia is a consequence of (or is ‘secondary’ to) brain damage; damage that may result from natal trauma, for example, and which will often cause other motor system abnormalities such as spasticity. The response of these patients to DBS is more variable. Because only dystonic movements are reduced by DBS, the presence of spasticity and other motor system abnormalities will limit any functional gain (Marks *et al*. 2009): the patients with secondary dystonia who respond best, therefore, are those whose functional capacity is inhibited mainly by dystonic movements. In order to improve the chances of a successful outcome, clinical teams offering DBS must be able to clearly diagnose dystonia, and to clearly delineate the extent to which it affects a patient's functional capacity.

In this article, we will explore the diagnostic activities of a multidisciplinary clinical team, the Paediatric Motor Disorder Service (pseudonym), providing DBS to children with secondary dystonia. Specifically this article will explore the activities of two team members, both physiotherapists, as they diagnose dystonia and determine the extent to which it impedes a patient's functional capacity. Many of the Paediatric Motor Disorder Service (PMDS) patients have severe forms of cerebral palsy, in which dystonic movements often co‐exist with spasticity, muscle weakness, and contractures. In these patients it is not unusual for both dystonia and spasticity to occur in the same regions of the body (‘mixed hypertonia’) and distinguishing the two becomes clinically difficult (Lebiedowska *et al*. 2004). Consequently, it is often unclear which motor sign is causing the patient the greatest discomfort or has the greatest impact on motor function, and thus whether DBS is an appropriate intervention. The PMDS's solution to this challenge is to rely on the embodied knowledge and tactile skills of the two physiotherapists (PTs). As we will see, the PTs carefully construct a diagnostic space, and within this space they use their own bodies to extract information from the body of the prospective DBS patient; information which is used to diagnose dystonia and thus decide whether or not to proceed with DBS. Using the PMDS as a case study, this article will argue that the corporal work of clinicians is an important component of many diagnostic processes and an important component of medical work more generally.

A recent emergence of literature in sociology has demonstrated that diagnosis is a useful focal point for examining not only the social dimensions of health and illness, but society as a whole (Jutel 2009, 2011, Jutel and Nettleton 2011, McGann and Hutson 2011). Authors have explored the social construction of diagnostic categories (Armstrong 2011, Salter *et al*. 2011), the consequences of diagnosis (Singh 2011, Trundle 2011, Willig 2011), and the diagnostic process itself (Schubert 2011). This last aspect of diagnosis, diagnosis‐as‐process, refers to the practices by which an afflicted individual's symptoms and signs obtain coherence in terms of existing disease classification systems. This may, of course, involve multiple investigations and trial and error, but the consequence is the production of useful knowledge that permits further clinical action. An examination of diagnostic processes has much to offer social scientists. Diagnosis provides an opportunity to explore various modes of clinical knowledge production, or the means by which the body, disease, and illness are rendered intelligible within clinical settings. Indeed, diagnostic processes provide an opportunity to explore a key problematic in the social sciences: how do actors generate and negotiate an understanding of themselves and the world within which they live? In contemporary healthcare, this ‘generation of understanding’ is increasingly mediated by specialist technologies such as MRI (Gardner 2014), but a great deal of diagnostic work still takes place within consultations that may only involve a patient and a clinician. In this article, we will draw attention to two aspects of clinical work involving the body that enable the ‘generation of understanding’ within such consultations. Using the PMDS physiotherapists as a case study, we will demonstrate that clinicians use their body as communicative apparatus within a carefully constructed material terrain, thus producing a diagnostic space, and we will demonstrate how, within this space, clinicians employ kinaesthetic skills, or an ability to engage in sensorial reflexivity, in order to obtain information. We argue that the knowledge required to undertake diagnostic practices is embodied (as well as having a cognitive dimension): clinicians possess a body that has learnt to be sensitive to, and moved by, a set of contrasts that many other bodies would fail to register. Importantly, we demonstrate that this embodied knowledge is activated by a diagnostic space: a carefully constructed terrain of material and semiotic elements that guide and channel the perceptual abilities of the clinician. By drawing attention to the embodied nature of diagnostic knowledge and the way in which such knowledge is activated by diagnostic spaces, this article makes an original contribution to the sociology of diagnosis. We argue that a ‘diagnosis’ can be conceptualised as emerging from an interaction in which space, the clinician‐body, and the patient‐body (or body‐part) mutually configure one another.

## The clinician‐body, space, and knowledge production in medicine

Scholars within medical sociology have tended to focus on the bodies of patients and those subjected to clinical discourses and practices, rather than the bodies of health professionals. Whilst this work is obviously of great importance to the sociological enterprise, its focus can give the impression that clinical knowledge is disembodied, and that clinical work is something that is done to bodies, rather than done via bodies. This article seeks to re‐address this balance by demonstrating the importance of the clinician‐body in diagnostic work, and by drawing attention to the importance of space in this corporal diagnostic work.

Sociological studies that explore the clinician‐body (as opposed to the patient‐body) can be roughly divided into two groups, each illustrating a different aspect of corporal work conducted by health professionals. First, there are studies that have explored what could be called the communicative body. Here, scholars have analysed the ways in which health professionals use their body to help encourage the compliance of patients within clinical interactions (Heath 1986, 2002, Brown *et al*. 2011, Måseide 2011). Heath (1986), for example, has explored the ways in which both clinicians and patients use their gaze and body language to indicate recipiency and to prompt the other to talk. Brown and colleagues (2011) have explored how clinicians use their bodies to convey information to patients, usually as a complement to verbal language, within the context of gynae‐oncology. For many of the patients included in the study, whether or not they trusted a clinician depended a great deal on the clinician's bodily actions and gestures. Particular forms of body movement and presentation inspired trust and confidence in patients, thus facilitating clinical practice. In a similar vein, Måseide (2011) noted how clinicians used their own bodies to help instruct patients how to use and move their bodies during examinations. Respiratory physiological examinations, for example, require patients to physically interact with technical equipment in a precise (and challenging) fashion. In order to encourage patients to do this, clinicians would often use bodily gestures in addition to verbal instruction. If this were done successfully, the interaction would generate a textual artefact (such as a note on the patient's medical records) that could subsequently be used to inform further clinical action. Måseide (2011: 297) illustrates that communicative body work is essential to ensuring the success of the examination, and suggests that clinical examinations can be seen as ‘mutually constitutive processes between various agents, bodies and body modes’. These studies illustrate, then, that the body is a key instrument for communicating with patients and ensuring that clinical interactions proceed ‘on script’. Importantly, these studies also illustrate that the production of clinical knowledge depends upon an interactionally‐generated, shared understanding, or the achievement of a meaningful clinical space. Communicative body work and verbal communication enable the production of such a space by endowing elements within it with symbolism and meaning.

The second aspect of corporal work that has been explored (Harris 2011; Schubert 2011) is what Moreira (2004) has referred to as sensorial reflexivity. Sensorial reflexivity refers to the sensing‐and‐acting habits acquired by health professionals via training and clinical experience; the learned perceptual skills and embodied dispositions that may be difficult to verbally articulate and communicate, but are nevertheless a vital component of clinical practice. Drawing on Merleau‐Ponty's work, Harris (2011) illustrates that conducting even the most routine, mundane clinical activities such as inserting a cannula requires a tactile competency that can only be acquired through bodily practice, and can easily be disrupted if, say, a new type of cannula is introduced. Several studies of surgeons and surgical training and practice have thoroughly explored this embodied sensing‐and‐acting (c.f. Moreira 2004, Hindmarsh and Pilnick 2007, Prentice 2007, Zemel and Koschmann 2014). Moreira, for example, notes that the surgeons in his study had, via training and clinical experience, carefully attuned their tactile and visual senses to particular phenomena within the fleshy bodies of their patients. Only by learning how to sensorially register these particularities within the patient could they then act upon them and thus conduct the surgery. As Prentice (2007) argues, surgeons are trained to embody schemes of perception and thought.

While the communicative body enables the production of a meaningful clinical space, the ‘sensing‐and‐acting’ clinical body is configured as such *by* a meaningful clinical space. This is cleverly illustrated in Moreira's study (2004): the sensing‐and‐acting abilities of surgeons are dependent upon a wider array of carefully arranged material and semiotic elements: patients are very carefully prepared before surgery so as to present specific body regions to the surgeon, and the operating theatre is precisely arranged in such a way that it enables, constrains, and directs the perceptual abilities of the surgeon towards particular attributes of the patient. Moreira points out, then, that both the surgeon and the patient are the subject of this wider surgical space: the patient's body is configured to present particular sensory affordances, while simultaneously the surgeon is configured as a particular sensing‐and‐acting surgeon‐body. In effect, the surgeon's learned, embodied schemes of perception are ‘activated’ by a network of material and semiotic elements that constitute the surgical space.

These studies of the communicative body and of sensorial reflexivity in surgical practice illustrate the spatially‐situated nature of the productive clinical body. Body gestures (along with verbal communication) laden a material environment with symbolism and meaning, and a meaning‐laden material space configures particular modes of sensing‐and‐acting within. We suggest, therefore, that the relationship between the knowledge‐producing clinical body, the patient‐body, and clinical space is one of mutual co‐constitution. Clinical knowledge emerges from an interaction during which clinical space, the clinician‐body, and the patient‐body are simultaneously configuring one another. In what follows, we use the diagnosis of dystonia within the PMDS as a case study to argue that the diagnostic process can be conceptualised in this way. Using the notions of communicative body work and sensorial reflexivity, we argue that diagnosis is a process whereby diagnostic knowledge – usually inscribed in a durable form such as a text – emerges from an interaction in which space, the clinician‐body, and the patient‐body (or body‐part) mutually configure one another. As we will see, the PTs within the PMDS actively produce, and are configured by, a diagnostic space.

The conceptualisation of diagnosis being proposed here has some similarities to science and technology studies (STS) work which has sought to highlight the artisanal, craft‐work nature of scientific activity (Knorr‐Cetina 1981, Lynch 1982, 1985, Latour 1987, Latour and Woolgar 1979). These studies illustrate how tacit knowledge and ‘sensing‐and‐acting’ transform local specificities (the built environment, laboratory apparatuses and materials) and formal rules (such as scientific protocols) into novel, explicit statements about the world. Embodied knowledge, in other words, is a vital component in a chain of transformations between forms of matter (raw materials and reagents), inscriptions (tables, graphs and diagrams) and explicit propositions that characterises laboratory work (Callon 1995). This STS work has also drawn attention to the materially‐situated nature of knowledge production. Laboratory instruments, for example, can be conceptualised as materialised, ‘reified theory’ (Bachelard in Latour and Woolgar 1986: 66): the consequence of black‐boxing particular sensing‐and‐acting practices within a machine. Laboratories themselves are constituted by physical demarcation of an interior controlled space, protected from unwanted intrusion and noise that could disrupt the sensing‐and‐acting activities of machines and bodies within (Henke and Gieryn 2008; Guggenheim 2012). In a similar vein, we will also draw attention to the materiality of diagnosis. We will illustrate that the diagnostic process requires a carefully constructed material terrain, which becomes a productive diagnostic space as it is laden with meaning via talk and communicative body work. This material and semiotic‐constituted diagnostic space enables the generation of what we refer to as momentary affects: temporarily‐induced patient‐bodily phenomena which are registered by (or affect) the senses of the clinician. The clinician then translates this momentary affect into explicit propositions which can be inscribed in notes, medical records, or some other form of durable text. Specifically, we will see that the process of diagnosing dystonia requires the physiotherapists to use communicative body work (in addition to talk) to prompt the patient to move and position their body within a material terrain in such a way that it presents opportunities to examine specific parts of their motor system. In order to take advantage of these opportunities, the physiotherapists employ their tactile sensibility to ‘feel’ spasticity, muscle weakness, contractures and dystonia. These sensations may then be translated into verbal articulations and/or recorded in notes which help inform a formal diagnosis.

This conceptualisation which we are outlining here puts embodiment at the centre of the diagnostic process, even in those contexts where diagnosis is heavily mediated by technologies. By doing this, we hope to provide another important counter to the Cartesian‐model of knowledge production which has tended to elide the body from accounts of knowledge production. And as we will discuss, this conceptualisation also has implications for the way in which we perceive the authority and power associated with diagnosis.

## Methodology

The data for this article were collected as part of a 12‐month ethnographic study of the Paediatric Motor Disorder Service. Data collection methods included interviews with team members (n = 12), observations of team meetings (n = 31) and observations of interactions involving team members, patients, and patients’ supporting family members (n = 6). The purpose of the fieldwork was to explore what Morlacchi and Nelson (2011) refer to as the learning‐in‐practice component of medical innovation: how, during day‐to‐day clinical activities, PMDS clinicians learned to integrate DBS technology into a clinical service for children and young people with dystonia. Accordingly, interviews were used to explore clinicians’ perspectives of the challenges associated with the integration of the technology. These were audiorecorded and transcribed. Observations of team meetings and interactions with patients were used to identify how these challenges manifested in day‐to‐day clinical practice, and how clinicians attempted to manage them. Observations were recorded in handwritten field notes, and all data were coded using NVivo9 software (QSR International, Melbourne).

Importantly, the data were collected using a material semiotics methodology (Law 2008; Gardner *et al*. 2011). A material semiotics approach assumes that human activity is not only culturally and historically situated, but also materially situated (Mol 1999). Non‐human entities (technologies, objects, the built environment) can facilitate, constrain, shape and transmute the activities of humans in ways that cannot be reduced to the activities of other humans (Latour 2005). Consequently, during observations we sought to describe the language and phrasing employed by participants, the material context in which interactions took place, and the way in which the physical bodies of participants interacted with each other and with other material elements.

Ethics approval was granted by a NHS Research Ethics Committee. Informed consent was obtained from all participants (PMDS team members, patients, and supporting family members) who were 16 years‐old or older. Assent was obtained from all participants younger than 16‐years of age, and informed consent was obtained from their supporting guardians. Age‐appropriate participant information leaflets were provided to patients, based on the format recommended by Alderson and Morrow (2011).

## Diagnostic tools: the GMFM and the musculoskeletal screen

The physiotherapists use two assessments to identify dystonia and determine its impact upon the posture and motor function of the patient: a manual musculoskeletal screening, and the ‘Gross Motor Function Measure’ (GMFM).

The GMFM was developed in the 1980s to assess children and young people with cerebral palsy. In order to conduct the examination, the client must attempt a range of tasks involving various ‘gross motor’ movements, such as lying, rolling, sitting, crawling, kneeling, running and jumping (Russell *et al*. 1989). As the client performs each task, the physiotherapists provide a score using a four‐point scale, where zero is ‘does not initiate’, and three is ‘completes’. From these individual task scores an overall score is calculated. Importantly however, as a client performs the tasks the physiotherapists are provided with the opportunity to interpret and diagnose the possible causes of gross motor function impairment. The resulting information can then be used to decide upon an intervention (Tieman *et al*. 2005). It is in this respect, as guide for intervention planning, that the GMFM is particularly useful for the PMDS.

Conducting the musculoskeletal screen requires the physiotherapist to engage in physical work with the patient. Particular manifestations of neurological pathology, for example, have a signature ‘feel’, but this is perceptible only if the patient's body is manipulated in a specific fashion (Reeves and Swenson 2008). Within the PMDS, the physiotherapists use musculoskeletal screening to physically detect and locate spasticity, muscle weakness, contractures and dystonia. Together, the musculoskeletal screen and the GMFM enable the PMDS physiotherapists to diagnose dystonia by differentiating it from other manifestations of neurological pathology, and to produce an overall picture of how each manifestation is impacting upon a patient's gross motor function.

In order to successfully conduct both the musculoskeletal screen and the GMFM, the physiotherapists use a range of tools and props. As the following section will illustrate, these tools and props constitute part of a material terrain which becomes a diagnostic space. In order to explore this in some detail, we will follow Carl, a 16 year‐old patient with secondary dystonia. Carl is accompanied by his mother, and the assessments are conducted by both PMDS therapists.

## Constructing a diagnostic space

The musculoskeletal screen and the GMFM take place in the hospital gymnasium. Importantly for the physiotherapists, the gymnasium is large enough with sufficient open space for the patient to perform various GMFM tasks (one task, for example, requires the patient to run ten metres in a straight line). The gymnasium also contains adjustable couches and benches, height‐adjustable desks, mats, several inflated balls of various sizes and an area that has been delineated with a pattern of floor markings.

Before Carl's assessment begins, the physiotherapists go about adjusting and arranging many of the objects within the gymnasium, creating a material spatial configuration in which the musculoskeletal examination and GMFM will be conducted. Some aspects of this configuration of material objects are prescribed by the GMFM manual and are therefore (ideally) uniform across all GMFM assessments. As the PT states:

The GMFM is quite defined about having something at waist height and defines what there is. Various height adjustable benches and tabletops and all those kinds of things [are required].

So, just prior to commencing Carl's assessment, one of the physiotherapists (PT1) asks him to stand next to the couch so that she can adjust it to his waist height. Carl is also asked to sit on the bench which is then altered so that his feet are placed squarely in the ground. Meanwhile the other physiotherapist (PT2) pulls two floor mats together and places them nearby, thus providing several metres of padded floor space. Some elements within the gymnasium have been permanently arranged so that they align with the GMFM manual. The most obvious example of this is the set of guiding floor markings, a series of lines of various lengths and a circle, which have been painted on the floor in the middle of the gym. These can be used to help guide the patient perform various GMFM tasks, such as walking in a straight line or jumping a specific distance. During an examination, then, a patient can simply be instructed to ‘jump from inside the circle to the line’. As PT1 states:

The idea [of the markings] is that there's quite set criteria in the test that the patient has to adhere to. For instance, if they walk along the line, they have to have their foot on the line, they can't step off. So we have the facility set up ready to go. Having them predetermined – it means we're all using the same marks each time … it also means all the professionals are using exactly the same test criteria to score the children on. So that's the advantage of those (PT1, interview).

These guiding floor markings, along with the adjusted bench and couch and the padded floor mats, constitute a partially‐standardised material terrain that will enable the production of clinical knowledge. As various STS scholars have argued, the material configuration of space is an active, structuring force in the production of knowledge (Gieryn 2002, Henke and Gieryn 2008, Guggenheim 2012). This ‘structuring force’ is, in part, a consequence of the capacity of the material terrain to prompt, guide and channel human activity in such a way that it presents various affordances for knowledge production and action, and it is a consequence of physical demarcation that shields activity within from noise, pollutants and intrusion (Gieryn 2002: 48). As we will see, the gymnasium terrain created by the PTs participates in configuring the body of the patient in such a way that his movement disorder is enacted as particular bodily effects, and it participates in configuring the PTs’ bodies in such a way that their perceptive skills are rendered sensitive to these effects. For this to occur, however, the patient must be prompted to move and position his body in particular ways, and this requires the achievement of shared understandings. This is achieved through communicative body work and verbal communication, which together laden the material terrain with meaning. The material terrain thus becomes a productive diagnostic space.

## Communicative body work

Carl's assessment begins with the GMFM. The role of the PTs is to ensure that Carl attempts to perform each of the tasks prescribed by the GMFM manual, within the diagnostic space they have created. While much of this is done via verbal instruction, the PTs also rely on their body as a means of communication. As Måseide (2011) has demonstrated, clinicians often use bodily gestures to help encourage the compliance of their patients during an examination; compliance that is necessary for the generation of sought after clinical information. Similarly, during the GMFM the PTs use both verbal and corporal communication to instruct Carl how he should position and move his body, often in reference to other material objects within the diagnostic space. Below is an example where the more junior of the two PTs (PT2) uses her body to instruct Carl on how to perform ‘task 41’, which requires moving from ‘prone’ to ‘4‐point, weight on hands and knees’. (The other PT watches and provides a score for Carl's attempt):

PT2 I want you to lie down on your front, flat on the mat facing me and then get up on all fours. Like this. [*She lies face down on the floor flat (prone)*,* and then picks herself up so that she is resting on her knees and hands (4‐point)*]

Carl [*Repeats the task without noticeable difficulty*]

In effect, the PT embodies a small part of the GMFM text: she performs it, with the intention that Carl will mimic her and do the same. Corporal and verbal communication are used together as a means of complementing one another throughout the assessment. In the process, bodily movements and words acquire specific meaning within the assessment. Indeed, verbal utterances are indexical to the PT's body movements and the material and discursive elements that constitute the diagnostic space. An example of this is the PT's instructions on how to perform ‘task 45’, which requires the patient to ‘crawl reciprocally forward for 1.8 m:

PT2 The first thing we are going to do is the commando crawl. Get down on that mat for me. You need to pull yourself along the mat and keep your body very low, to the end of the second mat *[which is approximately a distance of 1*.*8 m]*.

Carl *[Looks puzzled]*.

PT2 Okay, this is what I mean. *[She then gets down on the floor so that she is resting on her stomach and elbows*. *She then uses her elbows and knees to propel herself forward*.*]* Remember to keep low.

Carl *[Carl*,* without speaking*,* gets down on the floor and does this same with some difficulty]*

PT2 Remember to keep low!

Here ‘commando crawl’ and ‘keeping low’, descriptions that initially caused confusion, are equated with specific corporal form and movement; they acquire specific referents within the assessment, and in the process, material elements such as the mat acquire meaning. By using her body to complement verbal instructions, the PT is, in effect, participating in the production a meaningful semiotic world: the material terrain is interactionally‐transformed via an interplay of physical enactment and verbal instruction into a diagnostic space, which will subsequently enable the production of diagnostic knowledge.

A consequence of successful communication is that the patient will attempt to perform a series of GMFM‐prescribed body postures and movements. Importantly, these postures and movements are prescribed in relation to other material elements within the gymnasium: the patient is prompted to engage in carefully‐coordinated interactions with the entities that constitute the diagnostic space. Within these interactions, the patient's body is configured so that particular bodily phenomena are framed and amplified in such a way that they can be registered by the perceptual skills of the PTs. A good example of this are tasks 54 and 55, which require the patient to stand upright, holding on to a bench with one hand and lift the right foot off the ground, and then the left foot of the ground. PT2 provides instructions while PT1 makes a note of the score:

PT2 Now, stand next to the couch. Just stand there for 20 seconds, as still as you can.

Carl *[Stands next to the couch*,* but his shaking body makes it difficult for him to keep balance*. *Several times he has to adjust his feet to keep himself from falling]*.

PT1 How much effort does it require to stand still?

Carl It is hard to stand still. I can't stand still at all.

PT2 Now, put your hand on the couch, and try and see if you can lift the alternate foot.

Carl *[Does this with the left foot with some effort]…*

PT2 One, two, three, well done.

Carl *[Tries with the right foot*,* but has to place his second hand on the bench to stop himself from falling]*.

PT1 Carl, would you mind taking off your t‐shirt? I want to see what is happening with your spine.

PT2 [According to the GMFM manual] you get three goes on each foot. Try standing on it. One, two, three, four, five…

Carl This is the problem, when I try and stand on this [right] foot.

PT2 Your left foot moves around and throws you off balance, doesn't it.

Carl Yes.

Here, a GMFM‐prescribed body‐object interaction involving Carl, the bench and the flat surface of the floor has generated clinically‐relevant bodily phenomena: an otherwise obscured motor system abnormality is coaxed by the ensemble to manifest as a flailing foot and a twitching spine, phenomena that are registered by the PTs. These are momentary affects: temporarily‐induced patient‐bodily phenomena which are registered by (or affect) the senses of the clinician. Mol's (2002) notion of enactment is useful here. Within the GMFM‐prescribed ensembles, Carl's motor system abnormalities are enacted as an inability to carry out a prescribed task, or (as we will see further on) as specific tactile sensations. This enactment is collective, as it involves an arrangement of material objects, the compliant patient and the coordinating and attentive PTs; and it is also momentary, as it lasts only as long as these elements remain within a precisely arranged ensemble.

Importantly, aspects of this enacted motor system abnormality will be captured and translated into more durable modes of representation such as a text. During each GMFM‐prescribed movement and posture, a PT will take notes and provide a score for Carl's performance. As the following examples show, while the junior PT (PT2) instructs Carl, the other PT (PT1) watches carefully and provides a score for his performance on the GMFM score sheet. The first example, task 72, requires the patient to walk forward ten steps carrying a large object with two hands.

PT2 Okay, now I want you to walk holding this ball.

Carl *[Picks up the large*,* inflatable pink ball which has two small appendages on either side*,* and walks to the end of the guiding lines*. *Despite appearing a little unsteady*,* he does this without any obvious difficulty]*

PT1 Easy peazy! No problems there *[She scribbles a 3 on the GMFM score sheet*.*]*

In this case Carl's bodily movements, movements that entail negotiating other material elements within the gymnasium, are translated by the PT into a number (3) that is subsequently inscribed on the score sheet adjacent to the specific task number (72). At a later time, these scores and the accompanying notes will help inform the team's predictions on how Carl might respond to DBS. Here we see, then, that particular momentary affects have been translated into durable diagnostic inscriptions.

## Sensorial reflexivity

Once the GMFM is completed Carl and the PTs have a short break before beginning the musculoskeletal screen. By this stage, the PTs have a good idea of which particular areas of the motor system are causing difficulties for Carl, and this information is used to decide which areas of his musculoskeletal system will be screened. Carl's shoulders, pelvis, hips, knees and ankles will be screened for range of movement, muscle weakness, spasticity and contractures and this will, ideally, enable the PTs to decipher the presence of dystonic movements.

The assessment takes place on the adjustable couch. Throughout, Carl is instructed to adopt a number of body positions, depending on the particular aspect of the musculoskeletal system that is being screened. Much like the body‐object interactions of the GMFM, in each one of these positions the material form of the couch participates in moulding and supporting the patient's body in such a way that clinically‐relevant bodily phenomena are framed and amplified. And as with the GMFM, the compliance of the patient requires the interactional achievement of shared understanding. However unlike the GMFM, the musculoskeletal screen requires that PTs themselves become physically involved in the interaction. When testing for range of movement or smoothness of movement (which can be used to detect spasticity), the patient is instructed to remain passive, and the PT will support and move a part of the patient's body in a specific fashion. Standard techniques of musculoskeletal screening, outlined in various user manuals and learned by PTs as part of their training, describe how the PT should use their body in this way. For example, many examinations require the PT to apply ‘overpressure’ to a particular joint, which involves gently flexing or extending a joint beyond its usual range. While doing this, the PT is instructed to ‘be in a comfortable position’, use their ‘body weight or the upper trunk to produce the force, rather than the intrinsic muscles of the hand, which can be uncomfortable for the patient’, and in order to accurately direct this force, ensure that the their ‘forearm is positioned in line with the direction of the force’. All force should be ‘applied slowly and smoothly to the end of the available range’ (Ryder 2011).

In some cases the patient will take a more active role in creating momentary affects. When testing for muscle weakness, for example, a patient will be instructed to attempt to move a limb while the PT uses her own body weight to create resistance. Here is a specific example from Carl's assessment. Carl is seated upright on the couch, with his legs dangling off one end. The PT is exploring the muscle strength in his quadriceps:

PT2 Carl, I'm going to try and hold your feet down and I want you to try as hard as you can to extend your leg. *[She moves to the end of the couch and holds both his feet*,* one in each hand]*.

Carl *[Slowly extends his legs*,* with noticeable effort*,* while the PT exerts pressure]*

PT2 [Talking to PT1]: There is a bit of loss at inner range – I think it was the jerkiness.

PT1 That fits with the movement disorder.

This example also illustrates another purpose of the PT's body within the ensemble: to be momentarily affected. As various physical manipulations take place, the PT employs a carefully honed tactile sensibility to assess muscle strength, smoothness of movement, and movement range, which help to identify motor system abnormalities. Here then, the body‐body‐object ensembles are enacting motor system abnormalities as particular bodily sensations. During an interview, the junior PT described how particular abnormalities ‘feel’ when she is conducting a screen with a patient.

To see if a child or a young person has spasticity, you bend the knee very quickly. You'll suddenly get like a bony block, it will feel like a bony block but it's not a bony block, but it's like a catch. And then it will release and you will be able to bend it a bit further. Now that is spasticity. Whereas if you have the leg that's straight and you're about to bend it and you struggle to bend it throughout range, but you don't get one of these fast bends where you get a stop, then that's high tone … Sometimes you might get a leg and bend it and it's really floppy, so you would call that low tone. And then other times you'll try and bend a knee and you can't flipping bend it because the quadriceps are kicking in, which is dystonia, it's literally kicking in and stopping you bending [their knee]. It's completely rigid and you can't bend it.

Indeed, as the PT points out, various motor system abnormalities have their own ‘signature’ feel. This ‘bony block that is not a bony block’ sensation of spasticity, which can only be detected when the muscles are passively moved at high velocity, is often described as the ‘clasp‐knife’ response (due to the similarity with the rapid increase, and then rapid decrease, of resistance when closing a folding pocket knife) (Burke *et al*. 1970). Similarly, the difficulty in bending a knee through its range of movement due to high tone (or hypertonia) has been referred to as ‘lead‐pipe rigidity’. These are, of course, descriptions of temporary, collectively enacted bodily phenomena.

During Carl's assessment, then, the PTs are drawing upon a sensorial understanding of the motor system and its abnormalities. This tactile capability has been acquired during their professional training and has no doubt been honed in clinical practice. They have acquired a body that is tuned to particular sensorial affordances, just as the neurosurgeons described by Moreira (2004) have become sensitive to numerous visual and tactile sensorial differences within an operating theatre. To borrow Latour's (2004) parlance, the PTs possess bodies that have learnt to be affected by and moved by a set of contrasts that many other bodies would fail to register. And importantly, it is within a specific, material‐ and semiotic‐constituted diagnostic space that this sensorial capability is realised.

As with the GMFM, these temporarily generated bodily phenomena are translated into a more durable form. The following is from an examination which requires Carl to lie on his back, holding his knee in a flexed position while the PT splays his legs:

PT2 I got some resistance there, and the abductor switched on.

PT1 I think it is those intermittent jerks.

And during an examination of the range of motion of his left knee:

PT2 Ahh – there is a catch here.

Here, groups of words are used to articulate particular tactile sensations that, as a result, acquire a discursive existence as ‘intermittent jerks’, a ‘catch’, or ‘some resistance’. These utterances are then inscribed in text: both PTs jot down some of these utterances along with their interpretations (‘muscle weakness’ ‘spasticity’ and so on) in handwritten notes, so that by the end of the screening they have produced a document that provides a textual picture of various aspects of Carl's motor system and its abnormalities. There has been, therefore, a series of translations from bodily phenomena to utterance to text, in which the embodied knowledge of the PTs, their ability to physically mould and manipulate the patient, and their honed tactile sensibility, have been essential.

By the end of the GMFM and musculoskeletal screen the PT's have produced sufficient information to provide some sort of diagnosis. Based on their sensorial understanding of Carl's motor system, they declare that he does indeed have dystonic movements. These movements are detectable in regions of his upper body, his left leg, and he has dystonic posturing in his feet, all of which impair his gross motor function. But, the main problem for Carl, the PTs inform him, is muscle weakness around the pelvis.

PT1 Carl, the hip abductors and hip extensors are an issue. The main issues are around your pelvis, it is due to muscle weakness. Maybe we could teach you to do some exercise that could help you there.

This information is also recorded in Carl's medical notes. During a subsequent meeting, several PMDS clinicians use these notes to inform a prediction of how DBS may benefit the patient: Carl and his mother are told that it may improve his ability to perform tasks that involve using his upper body, but that it is unlikely that it will directly improve his stability while standing and walking.

## Discussion and conclusion

The diagnostic corporal skills of the physiotherapists are an important component of the Paediatric Motor Disorder Service. The knowledge gathered during the Gross Motor Function Measure assessment and the musculoskeletal examination help team members to predict how a patient will respond to deep brain stimulation, thus enabling them to identify which patients are suitable candidates for deep brain stimulation. There are no technological methods for doing this. While MRI and PET may render central nervous system lesions visible, the images generated cannot be used to determine whether such lesions will result in dystonia or spasticity. The physiotherapists, however, possess a somatic awareness of how to conduct a successful diagnosis: they have acquired bodies that can register, and be moved by, the subtle differences between various types of motor disturbances. A successful diagnosis depends upon their ability to transform a material terrain into a meaningful diagnostic space, and their ability to perceive and register contrasts that many other bodies would be insensitive to.

The diagnostic knowledge of the physiotherapists, then, is corporal as well as cognitive. Drawing on the work of Merleau‐Ponty (2002), Crossley (2001) describes such corporeal knowledge as ‘embodied know‐how’. It is an embodied understanding – aspects of which may be pre‐reflexive – of how to move the body and register the surrounding world: a ‘perspectival grasp upon the world from the ‘point of view’ of the body’ (Crossley 2001: 102) that is inseparable from practical action and enables reflective thought. The physiotherapists possess a perspectival grasp of the motor system from the ‘point of view’ or their own physical bodies. The description of ‘clasp‐knife’ sensation that characterises spasticity (Burke, Gilles, and Lance 1970) is an example of this. By drawing attention to people's understanding of how to move the body and register the surrounding world, Crossley provides a contrast to the Cartesian model of knowledge production which has tended to dominate Western thought (Merleau‐Ponty 2002). According to the Cartesian model, knowledge is the domain of the mind: it is explicitly present in consciousness and it is (ideally) the product of reflective, sceptical and reasonable deliberations (Shusterman 2012). The effect of Cartesian dualism is that the role of the body and bodily‐sensations have largely been elided from accounts of knowledge production, scientific endeavour and medical progress. Knowledge, in other words, is portrayed as disembodied, ‘abstract, universal and placeless’ (Henke and Gieryn 2008: 343). By drawing attention to the corporal nature of diagnostic work, we aim to disrupt Cartesian‐like accounts of diagnosis and medical practice more generally in which the patient body is objectified by disembodied medical discourse.

In this regard, we build on a small collection of work that has explored the careful craft work involved in diagnosis (Buscher *et al*. 2010), and the embodied nature of diagnosis work more specifically (Moreira 2006, Goodwin 2010, Schubert 2011). Both Goodwin and Moreira, for example, draw attention to the importance of clinicians’ kinaesthetic skills in providing important diagnostic knowledge that supplements technology‐derived diagnostic information. Goodwin (2010) notes that anaesthetists use their sense of touch to acquire knowledge of aortic aneurisms that is important for the subsequent care of the patient, and Moreira (2006) illustrates how neurosurgical practice is guided by a neurosurgeon's own touch‐derived diagnosis of a patient's blood pressure, combined with the more formally‐derived sphygmomanometer measurement provided by the anaesthetist. The case studies provided by these authors illustrate that embodied diagnostic knowledge is an essential element of diagnosis, even in contexts where a great deal of diagnostic work has been ‘delegated’ to technology. In a similar vein, Schubert (2011) examines the relationship between diagnostic technologies and embodied skills, illustrating that the latter are configured by diagnostic tools such as the stethoscope. It is by working with tools that clinicians acquire many of the embodied perceptual skills that render them sensitive to otherwise indiscernible elements of the patient. Such tools prompt clinicians to acquire an embodied diagnostic knowledge, without which the tools would be useless and the patient would remain ‘unknowable’. Schubert (2011: 856) argues that diagnostic tools such as the stethoscope form part of ‘diagnostic ensembles in which bodies, tools, and knowledge are mutually configured’.

We build upon this work on embodiment in diagnosis by illustrating the importance of a carefully constructed diagnostic space, of which tools such as the stethoscope may be one component. Spaces like the gymnasium in which the GMFM and musculoskeletal screen took place are constituted by material elements which, through communicative body work and talk, become meaningful diagnostic spaces. These spaces prompt particular momentary affects: the patient‐body is configured in such a way that it generates clinically‐relevant phenomena, while the clinician is configured so that they are rendered sensitive to, and can act upon, these phenomena. These phenomena can then be translated into diagnostic information as explicit propositions and/or a text of some sort. Diagnostic space, then, is analogous to the carefully constructed laboratory spaces in which scientific knowledge is produced (Latour and Woolgar 1979; Latour 1987). The knowledge produced by such laboratories is not simply the product of the rationalistic cognitive deliberations of scientists and laboratory technicians (Henke and Gieryn 2008). Rather, it emerges from and is shaped by interactions involving material objects and instruments, reagents and texts, fleshy bodies, and the culturally‐mediated embodied dispositions and reasoning activities of individuals. We suggest that all diagnostic processes can also be conceptualised in a similar way: that diagnosis can be conceptualised as a process whereby diagnostic information emerges from a mutually co‐constitutive interaction involving bodies or body parts and diagnostic space.

Obviously the specific form of the elements involved within such interactions and their relative influence on the ‘diagnosis’ will differ greatly between diagnostic procedures. According to this conceptualisation, diagnostic technologies can be seen as a consequence of ‘black‐boxing’ a portion of diagnostic space. A flow cytometer, used to help diagnose leukaemia by counting white blood cell‐types, is a good example: the device casing and internal componentry are arranged in such a way to prompt and channel certain interactions within (a solution contain cells is passed through a laser), while reducing noise and unwanted ‘outside’ influences. These shielded interactions generate momentary phenomena (a fluorescing white blood cell and the fleeting deflection of the laser), which are registered by the carefully arranged detectors and are then translated into a durable inscription (the deflection pattern). Of course the successful utilisation of a flow cytometer requires it to be immersed in a wider set of material‐semiotic relations, or a ‘biomedical platform’ (Keating and Cambrosio 2003) involving other machines, reagents, texts and laboratory technicians with particular cognitive and tacit knowledge.

Thinking about the diagnostic process in this way has several sociologically‐relevant implications. First, many aspects of an individual's corporal knowledge, and their ability to produce and respond to particular spaces, will be collectively‐shared with other individuals, while some aspects will be acquired during professional training and practice and will thus be specific to particular professional groups (Crossley 2001). Indeed, such similarities and differences between corporal work are one basis for social and professional divisions. Within healthcare, some corporal diagnostic knowledge may be shared between clinical professions and medical specialists (neurologists, for example, are also trained in how to perform musculoskeletal screens for dystonia and spasticity), whereas other corporal diagnostic knowledge will be profession‐specific. We suggest that a useful future avenue of research would be to explore the way in which professional divisions between embodied knowledge – and the spaces within which it is deployed – are reified, disrupted or adapted in interdisciplinary clinical contexts. Such studies can shed light on some of the ways in which the move towards interdisciplinary work in some areas of healthcare (such as regenerative medicine) may be reconfiguring biomedical practice and knowledge.

Second, conceptualising diagnosis in this way also has implications for how we understand power and authority in diagnosis. Power has often been understood in terms of an agent's capacity to control other elements; an agent's capacity to bring about a state‐of‐affairs that enables them to achieve certain aims. Similarly, the making of a diagnosis is often seen as an exercise of clinicians’ power. They are formally endowed with the ability to control particular elements; to define the patient in such a way that permits or hinders access to treatment regimes. This is the social power of diagnosis (Jutel 2011; Jutel and Nettleton 2011). However, by attending to the mutually‐constituting relationship between the diagnostic body and diagnostic space, we are drawing attention to another element of this ‘power to diagnose’. On the one hand, a clinician's power is predicated on the ability to create a diagnostic space, to generate shared understandings with the patient and thus secure their compliance. This requires then, that the clinician exercises a degree of control – they must control their own bodily and verbal action so that they can compel the patient to act according to script. Yet, on the other hand, the power to diagnose also derives from possessing a body that has learned to be sensitive to, and affected by, the space within which it is situated. If the body is not sufficiently sensitive, then there is no momentary affect and there is no action. Moreira (2004) has made this point in regards to the ‘authority’ of a surgeon: a surgeon's power, he argues, derives from their carefully‐honed ability to be moved by, and respond to, the affordances presented by the socio‐technical network of the surgery. This requires that they temporarily remove themselves from other ‘disruptive’ networks and succumb (to a degree) to the elements that constitute the surgical space. From this perspective, a clinician's ‘power to diagnose’ may certainly derive from their formal position within particular institutions, but it is also predicated upon a body that can configure, and be configured by, particular forms of space.
